# Examinations for the Degree of D. D. S., in the Ohio College of Dental Surgery

**Published:** 1867-03

**Authors:** 


### Editorial.

### EXAMINATIONS FOR THE DEGREE OF D. D. S., IN THE OHIO COLLEGE OF DENTAL SURGERY.

It is probable that a few still labor under the impression that collegiate examinations are merely formal, and that the conferment of the degree is a matter of course. This is to be inferred from the fact that, every session, the faculty is besieged by those who wish to graduate, without having the prescribed requisitions, they taking it for granted that, as a matter of course, they can pass a satisfactory examination, even though it may be well known to the faculty that they would ignominiously fail, if submitted to the test. Two classes so press their claims on the faculty—those of a few months' study, and those who, though long claiming to be dentists, have not studied at all. The former are generally cured by one course of lectures; the latter would not be cured though "brayed in a mortar."

The proprietors and patrons of the Ohio College may desire to know something more than they do of the mode and extent of the examinations. This thought has led to the writing of this article.

Each candidate was subjected to a verbal examination by the whole faculty, on the subject of his thesis. Afterward, each professor submitted a series of questions or propositions, to be answered in writing, the answers to be written in the lecture room, in his presence, without reference to text books, or consultation with classmates. And to be successful, correct answers to two-thirds of the questions from each professor were required. This is more rigid than the requirement of last year, two-thirds of the aggregate being then demanded. It is not probable that a more rigid rule will be recommended by the friends of the institution; and that the questions are not trivial and formal will be recognized when they are read. Hence, they are subjoined, with the statement that no examination was made on chemistry, as the course was taught by different teachers, the professor of that department being absent by illness.

ANATOMY.

1. Give the muscles of shoulder joint.

2. Describe the superior maxillary bone—situation, articulations, surfaces, processes, etc.

3. Where is sphenoidal fissure? what does it give passage to?

4. Give the alimentary canal—its divisions, etc.

5. What nerves supply the special sense of taste? what nerve the tongue with motion?

6. What nerves supply the eye with sight and motion?

7. What are the coverings of oblique internal hernia?

8. Give the situation, lobes, fissures and ligaments of the liver, together with its offices, and where it receives its blood from?

9. Give the muscles of mastication.

10. Give the fœtal circulation.

11. Give the bones of the head and face.

12. Give the openings into the pharynx.

HISTOLOGY, PHYSIOLOGY AND PATHOLOGY.

1. Describe a cell and its contents.

2. What is the cellular arrangement in the various kinds of tissue?

3. Name the fluids of the mouth, the sources of their supply and their characteristics.

4. Enumerate the glands of the mouth, both salivary and mucous, and describe their anatomical structure and situation.

5. What portions of food are digested in, and absorbed by the coats of the stomach, and what by the intestines?

6. Name the secretions employed in the process of digestion, in the order in which they are furnished, and the office of each.

7. What is the composition of the blood?

8. Give the specific constituents of the plasma.

9. Give the circulation of the blood.

10. What change does the blood undergo in the lungs?

11. Describe the embryonic tooth from its earliest inception to the point of calcification.

12. State the order and the time of the eruption of the deciduous teeth.

13. Same of the permanent teeth.

14. What are the soft and what the hard parts of a tooth; and which, if either, is true bone?

15. Name the salts which compose the inorganic portions of teeth, and their relative proportions.

16. Describe the structure of the skin.

17. Describe the glands of the skin, and their secretions.

18. What are the various causes producing odontalgia?

19. Describe the tumors which occur in the mouth, designating the more frequent and the more rare.

20. How are the different varieties removed?

21. Describe the different diseases to which the maxillary sinus is liable.

22. Describe, in detail, the treatment of alveolar abscess.

### OPERATIVE DENTISTRY AND HYGIENE.

1. How is decay of the teeth produced? What is the principle?

2. What agents operate as exciting causes?

3. What are pre-disposing causes of decay of teeth?

4. Can any thing be accomplished for the arrest of dental caries, either by general or local treatment, otherwise than by filling? And if so, how much, and in what way?

5. Why is decay arrested sometimes, without the aid of the dentist?

6. Why does filling teeth arrest decay?

7. What more can be done than filling, for the arrest and prevention of decay?

8. What are the requisite qualities of materials for filling teeth?

9. What are the variations found in sensitive dentine?

10. Upon what do these variations depend?

11. What is the nature of this affection?

12. In filling teeth, what are the points to be observed, to secure success?

13. Give the successive steps in filling a simple crown cavity in a superior molar. Also, in filling a proximal cavity of a superior central incisor, both with gold, (each one selecting his own method of using it.)

14. What principles are involved in the treatment of dental periostitis?

15. What are the successive steps in the formation of alveolar abscess?

16. Describe the process of absorption, as exhibited in living organic tissue.

17. By what means is lost tissue restored?

18. Describe the process of granulation?

19. In what does a hemorrhagic diathesis consist?

20. What is the treatment for alveolar hemorrhage?

MECHANICAL DENTISTRY AND METALLURGY.

1. What are the best materials for taking impressions, and why?

2. What are the properties required for metallic model and counter-model, and what metals are the best?

3. How many sets of dies are necessary for the perfect swagging of metallic plates, and why?

4. What are the best metals for base plates, for artificial teeth, and qualities requisite?

5. Why are the base metals objectionable, and what are the bad results that may follow their use?

6. What are the objections to use of clasps in securing artificial fixtures?

7. What is the lowest standard fineness of gold admissible, and what objections to the lower standards?

8. What should be the relative fineness of the solder and the plate?

9. What are the alloys in gold coin, and how removed to make pure gold?

10. What are the agents used for removing the impurities and alloys from gold filings, and how used?

11. What are the alloys in silver coin, and how removed to make pure silver?

12. What flux is used when soldering metals, and why necessary?

13. What agents are used for pickling after soldering, and why necessary?

14. What are the advantages, and what the disadvantages in rubber for base for dental substitutes?

15. What are the objects to be gained by artificial teeth?

### CLINICAL DENTISTRY.

1. What are the various advantages in the use of the wedge in operating?

2. Methods of applying and changing napkins and compresses while filling.

3. Why is the prolonged use of the mallet less irritating to the tooth and its surroundings than hand force?

4. What is Therapeutics?

5. What are the effects of counter-irritants in inflammatory diseases?

6. Dental medicines—their properties and preparation.

7. What is a poison?

8. The specific effect of creosote in the sac of an abscess.

9. The systemic symptoms of poisoning by arsenic.

10. The antidotes for arsenic.

11. The treatment for *cancrum oris.*

12. The best method of testing the strength of the heart's action before administering chloroform.

13. The best position for the patient while using anæsthetics.

14. Epulis—and treatment.

15. The best mode of treating external dental fistulas and maxillary abscesses.

16. The treatment of the antrum in cases of tumors or polypus.

17. Tempering chisels, fine excavators, and canal drills.

18. Give the differential process of securing the requisite temper for the various shapes and sizes of pluggers and burnishers.

So much for the questions. The answers were examined in a faculty meeting, and freely discussed. The whole number of questions is 86. The correct answers of those who passed, range from 71 to 83, the average being about 91½ per cent. We believe the friends of the faculty and Trustees will approve of the award. A young man, of "good moral character," who can, without time for deliberation or opportunity for consultation, correctly answer 91½ per cent. of the above, ought to receive the honors of the institution, especially when he has previously written a good thesis, and successfully defended it before the faculty in a verbal examination. Such being the record of the late class, its members are cordially commended to the profession as brethren "in full communion." W.

